# Calcium-tin alloys as anodes for rechargeable non-aqueous calcium-ion batteries at room temperature

**DOI:** 10.1038/s41467-022-31261-z

**Published:** 2022-07-04

**Authors:** Zhirong Zhao-Karger, Yanlei Xiu, Zhenyou Li, Adam Reupert, Thomas Smok, Maximilian Fichtner

**Affiliations:** 1 Helmholtz Institute Ulm (HIU) Electrochemical Energy Storage, Helmholtzstr. 11, D-89081 Ulm, Germany; 2 Institute of Nanotechnology, Karlsruhe Institute of Technology (KIT), P.O. Box 3640, D-76021 Karlsruhe, Germany

**Keywords:** Batteries, Energy storage, Nanoscale materials, Energy

## Abstract

Rechargeable calcium batteries possess attractive features for sustainable energy-storage solutions owing to their high theoretical energy densities, safety aspects and abundant natural resources. However, divalent Ca-ions and reactive Ca metal strongly interact with cathode materials and non-aqueous electrolyte solutions, leading to high charge-transfer barriers at the electrode-electrolyte interface and consequently low electrochemical performance. Here, we demonstrate the feasibility and elucidate the electrochemical properties of calcium-tin (Ca–Sn) alloy anodes for Ca-ion chemistries. Crystallographic and microstructural characterizations reveal that Sn formed from electrochemically dealloying the Ca–Sn alloy possesses unique properties, and that this in-situ formed Sn undergoes subsequent reversible calciation/decalciation as CaSn_3_. As demonstration of the suitability of Ca–Sn alloys as anodes for Ca-ion batteries, we assemble coin cells with an organic cathode (1,4-polyanthraquinone) in an electrolyte of 0.25 M calcium tetrakis(hexafluoroisopropyloxy)borate in dimethoxyethane. These electrochemical cells are charged/discharged for 5000 cycles at 260 mA g^−1^, retaining a capacity of 78 mAh g^−1^ with respect to the organic cathode. The discovery of new class of Ca–Sn alloy anodes opens a promising avenue towards viable high-performance Ca-ion batteries.

## Abstract (web summary)

The key challenge for rechargeable Ca batteries originates from the severe passivation of the calcium metal anode in electrolyte solutions. Here, the authors demonstrate the feasibility and elucidate the electrochemical properties of calcium-tin (Ca–Sn) alloy anodes for rechargeable Ca batteries.

## Introduction

Growing concerns about the mid- and long term availability of certain raw materials such as cobalt and nickel as well as lithium (Li) to meet demands for global production of Li-ion batteries have motivated researchers to search for alternatives to Li-based electrochemical energy-storage systems^1,2^. New battery chemistries using earth-abundant materials could provide new pathways towards economic and sustainable energy storage solutions especially for large-scale stationary applications^3,4^. Considerable efforts are being devoted to developing energy-dense and high-performance post-Li battery systems based on sodium-ion (Na-ion), potassium-ion as well multivalent metals such as magnesium (Mg), zinc (Zn), calcium (Ca) and aluminum (Al)^5–8^. Multivalent ions are capable of transferring two or three electrons per ion, holding the promise of a two- or three-fold increase in gravimetric capacities as compared to monovalent Li-ion and Na-ion batteries. Moreover, in contrast to Li or Na, the low-tendency of dendrite formation of Mg and Ca metal anodes could offer the opportunity to improve the energy density and safety of the batteries.

Ca is the fifth most abundant element in the earth’s crust with an equal global resource distribution^2^. On the standard hydrogen electrode (SHE) scale, Ca has a lower reduction potential (‒2.87 V_SHE_) compared to Mg (‒2.37 V_SHE_) and Al (‒1.66 V_SHE_), and is close to that of Li (‒3.04 V_SHE_)^9–13^. Moreover, Ca offers a high volumetric capacity of 2073 mAh cm^−3^, which is at the same level as Li^6^. While Mg and Al metal anodes have higher theoretic volumetric capacities, their relatively less negative reduction potentials lead to limited cell voltages compared to Ca batteries^6^. The cell voltage and energy density of Ca batteries is potentially comparable with Li-ion batteries, and yet higher than Mg and Al batteries^5,6,11^. In addition, Ca^2+^ ion is larger than Mg^2+^ ion (effective ionic radii of 1.0 Å for Ca^2+^ and 0.72 Å for Mg^2+^)^14^ while carrying the same charge which may allow faster ion diffusion kinetics in solid electrode materials due to the greater softness of the Ca-ion. Thus, rechargeable Ca batteries could provide higher power density over other multivalent metal ions based battery systems. However, the research and development of Ca batteries faces multiple challenges, including the lack of efficient electrolytes and feasible cathode materials enabling reversible Ca-ion intercalation^12^. Additionally, the strong reducing power of Ca may induce electrolyte decomposition, forming ionically insulating layers on the metal anode^15,16^. Electrolytes of calcium tetrafluoroborate Ca(BF_4_)_2_ in carbonate solvents were found to be capable of conducting Ca-ions at an elevated temperature (>75 °C)^10^. Concurrently, identifying a suitable cathode is still fundamentally restricted by high kinetic barriers for ion transport at the Ca anode-electrolyte interfaces and insufficient electrochemical stability of electrolytes^12,15,17^. Some potential cathode materials could only be examined with specially designed cell setups (for example, using activated carbon cloth as the counter electrode) and veritable full-cell tests with these cathodes have not been validated^18–20^. Very recently, calcium tetrakis(hexafluoroisopropyloxy)borate (Ca[B(hfip)_4_]_2_) based electrolyte has been demonstrated, which exhibits electrochemical properties in terms of high efficiency (~80%) for reversible Ca deposition, high oxidative stability (4 V), high ionic conductivity (~8 mS cm^−1^) at room-temperature (25 °C), and good chemical stability under ambient conditions^21^. Meanwhile, with the advancement of the electrolytes, some developmental progress of Ca metal based full cells has been made^22–26^. However, severe passivation on Ca anode has been found to be one of the main causes for their unsatisfying cycling stability. Further research efforts on understanding interfacial chemistry and designing suitable anode-electrolyte interphase are therefore critical for realizing viable Ca-based batteries^27^.

In this respect, employing an alloy type of anode may offer a new path for achieving viable battery performance. Attempts to use an electrochemically calciated tin (Sn) as an anode has been made, where the Sn electrode exhibited an initial discharge capacity of 40 mAh g^−1^ in Ca(PF_6_)_2_ electrolytes^28^. Electrochemical calcination of (Sn) has also been proven in hybrid or dual-ion cell configurations^18,19^. In particular, the dual-ion system with a graphite electrode and calcium hexafluorophosphate Ca(PF_6_)_2_ based electrolyte exhibited stable cycling performance with the capacity retention of 95% after 350 cycles, where Ca_7_Sn_6_ was identified as the reversible active phase^19^. High-throughput DFT calculations have also suggested numerous Ca alloys as potential anode candidates^29^. The feasibility of calcium-silicon alloy (Ca–Si) anodes has been examined using CaSi_2_ǀǀCa cells with the electrolytes of Ca(BF_4_)_2_ in carbonate solvents at 100 °C, indicating that only decalciation process of CaSi_2_ could be confirmed^30^. However, it is worthy of mention that the employment of the conventional half-cell setup could lead to undervaluation of the electrode properties due to the severe passivation on the Ca metal counter electrode in the electrolytes. In this work, we propose a holistic approach by constructing a full cell with a kinetically favored organic cathode (1,4-polyanthraquinone) and a Ca–Sn alloy anode to circumvent the obstacle originating from passivation of the Ca metal anode and enable the redox chemistries on the anode and cathode simultaneously. Additionally, an electrolyte with favorable electrochemical properties is essential to ensure sufficient Ca^2+^ ion mobility and charge transfer at the electrode interfaces for enhanced battery kinetics. Herein, we present the electrochemical performances of the full cells comprised of the 1,4-polyanthraquinone (14PAQ) based cathode and a Ca–Sn alloy based anode in combination with the electrolyte of Ca[B(hfip)_4_]_2_ in dimethoxyethane (DME). Using X-ray diffraction (XRD) microscopic and scanning electron microscope (SEM) techniques, we also characterized the electrochemical calciation and decalciation of the Ca–Sn alloys, finding that Sn formed in the initial electrochemical dealloying process of the Ca-rich alloy transform to CaSn_3_ in the subsequent alloying process and this new phase is capable of reversible calciation/decalciation. We propose Ca–Sn alloys as potential anodes for Ca-ion batteries and demonstrate their feasibility with Ca-ion full-cell prototypes.

## Results and discussion

**Fig. 1:**
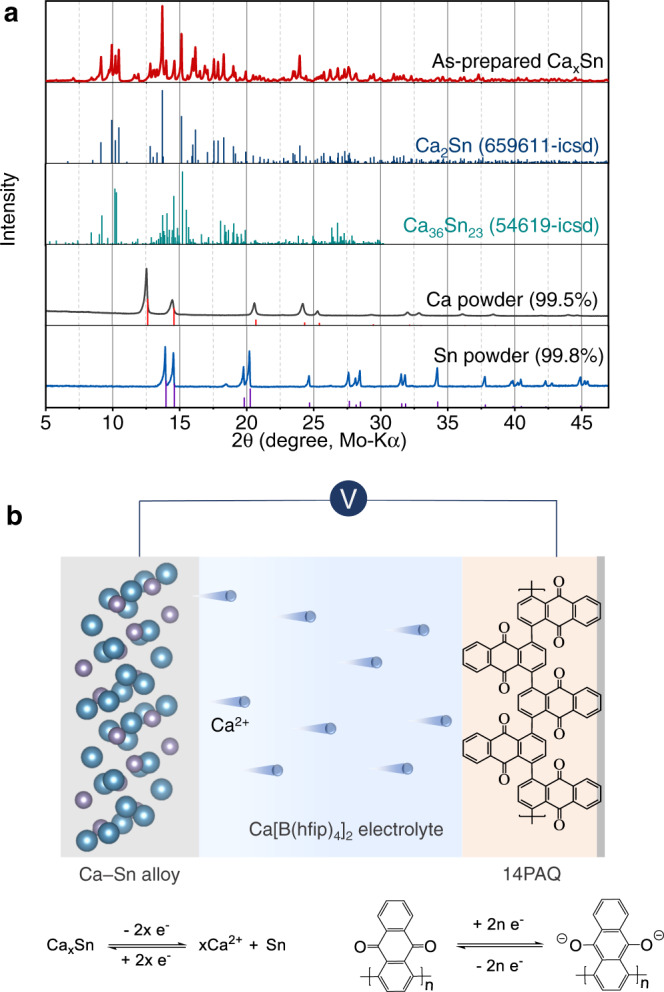
Characterization of the Ca–Sn alloy and the cell configuration with the Ca–Sn alloy anode. **a** XRD patterns of the as-prepared Ca–Sn alloy and the reference patterns, **b** Schematic illustration of the quasi-full cell configuration with the Ca–Sn alloy anode and 14PAQ cathode and the working principles.

Ca_2_Sn, with the richest Ca content among the Ca–Sn alloys, has a theoretic capacity (903 mAh g^−1^ based on Sn) and low decalciation/calciation potential (0.53 V *vs* Ca)^29^. Therefore Ca_2_Sn was primarily targeted in this study. The desired alloy was prepared by heating the mixture of calcium and tin powder at an appropriate ratio under Ar atmosphere at 900 °C. 10 wt.% Ca in excess to the stoichiometry for Ca_2_Sn was weighted to compensate for evaporation loss. XRD patterns of the as-prepared alloy shown in Fig. 1a indicated a mixture of at least two phases. Zintl phase Ca_2_Sn was identified as the dominate phase, where the main peaks at 13.7°, 15.1°, 16.1° and 17.1° correspond to the (211), (013), (020) and (004) planes of the orthorhombic Ca_2_Sn structure, respectively. Ca_36_Sn_23_ or/and Ca_32_Sn_21_ could be co-existing phases in the sample as they are closely located in the phase diagram and have very similar diffraction patterns^31,32^. (Supplementary Fig. 1 shows the related reference patterns.) Hence, as-prepared alloy was denoted as Ca_x_Sn in the following discussion. Initially, several attempts to electrochemically calciate micrometer-sized pure Sn or decalciate the as-prepared Ca–Sn alloys in a common half-cell configuration using a Ca metal pellet as the counter electrode were unsuccessful as shown in Supplementary Fig. 2. The ex-situ SEM analysis of the Ca electrodes in these cells revealed coarse surface and some crater-like spots that could be the electrochemically reactive sites (Supplementary Fig. 3), demonstrating the severe passivation of the Ca metal in the electrolyte solution. In contrast, the symmetric cells with the Ca–Sn alloy electrodes showed reversibly electrochemical process with a low voltage polarization (<0.25 V at 0.1 mA cm^−2^, over 100 h) (Supplementary Fig. 4). Therefore, in this study we employed a quasi-full cell configuration with an organic cathode to examine the feasibility of the Ca–Sn alloy anodes for Ca-ion batteries.

Polymer materials based on enolization redox chemistry has been proven to be suitable for reversible Mg-ion storage at relatively high current rates^33–36^. Herein, 1,4-polyanthraquinone (14PAQ) was chosen as the model cathode material and coupled with the Ca–Sn alloy electrodes. The Ca[B(hfip)_4_]_2_/DME electrolytes were employed for the cell assembly. The cell configuration and working principles are illustrated in Fig. 1b. Both electrodes were prepared by pressing the composite material onto a metal mesh with a diameter of 10 mm. The mass loading of the active alloy and 14PAQ is ~12 mg cm^−2^ and ~1.5 mg cm^−2^, respectively. In this work, the electrochemical properties of the Ca–Sn alloy anodes were qualitatively evaluated with respect to the 14PAQ positive electrode and, unless otherwise noted, all specific capacities and specific currents are calculated on the basis of the active loading of 14PAQ.

**Fig. 2:**
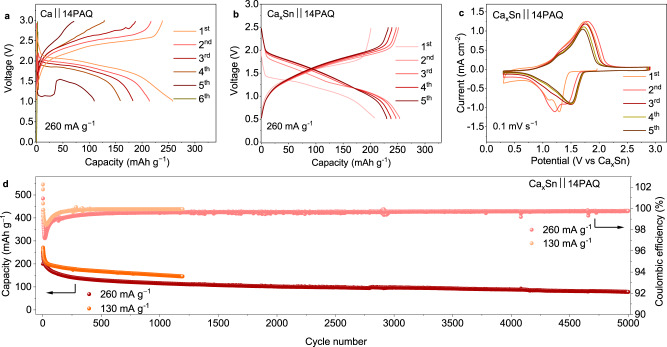
Electrochemical performance of Caǀǀ14PAQ and Ca_x_Snǀǀ14PAQ cells at 25 °C. Galvanostatic discharge/charge profiles of **a** Caǀǀ14PAQ, **b** Ca_x_Snǀǀ14PAQ cell with the Ca[B(hfip)_4_]_2_/DME electrolytes at a specific current of 260 mA g^−1^. **c** CV curves of the 14PAQ electrode using Ca_x_Sn as both counter and reference electrodes at a scan rate of 0.1 mV s^−1^. **d** Cycling performance of the Ca_x_Snǀǀ14PAQ cell at 130 mA g^−1^ (0.5 C) and 260 mA g^−1^ (1 C), respectively.

Galvanostatic cycling tests were carried out in a cell voltage range of 0.5–3.0 V for Ca-based cells or 0.5‒2.5 V for Ca–Sn alloy based cells at an appropriate current rate (1 C = 260 mA g^−1^, based on the two-electron redox reaction of the anthraquinone unit of the active material). Initially, we assembled Caǀǀ14PAQ cells as conventional half-cells with a Ca metal anode and the borate electrolyte; they exhibited a discharge voltage of ~2.05 V and capacity of 253 mAh g^−1^ in the first cycle (Fig. 2a). However a fast capacity fade and voltage degradation were observed (within 6 cycles), which were most likely caused by the passivation on the Ca metal anode.

When a Ca_x_Sn anode was coupled with the 14PAQ cathode, the Ca_x_Snǀǀ14PAQ cells exhibited distinctly different electrochemical features compared to the Caǀǀ14PAQ cells. As shown in Fig. 2b, the galvanostatic discharge/charge profiles display a cell voltage of about 1.8 V and an initial capacity of 248 mA h g^−1^ (in the 3rd cycle) at a specific current of 260 mA g^−1^ (1 C), which is about 95% of the theoretical value. The enolization redox chemistry of 14PAQ in the Ca-based batteries as shown in Fig. 1b was confirmed by ex-situ IR spectra in this study (Supplementary Fig. 5), which is similar as that of the Mg battery systems^33,35,36^. The cyclic voltammograms (CV) as depicted in Fig. 2c present one broad reduction peak at 1.29 V with a shoulder peak at 1.1 V in the first anodic scan and a main oxidation peak at 1.75 V in the reverse scan, which corresponds to the reversible enolization reactions of 14PAQ. The small shoulder peaks in the CV curves could be ascribed to the stepwise reactions with different accessibility of Ca^2+^-ions to the carbonyl groups of the polymer matrix. In the following scan cycles, the reduction peak shifted to ~1.50 V, implying an activation process in the initial cycles. The potential difference between the reduction and oxidation peaks decreased to 0.23 V after 20 CV cycles, reflecting the high redox reversibility of 14PAQ in the Ca–Sn based system. The CV results are consistent with the differential capacity diagrams i.e. dQ/dV plots derived from the discharge/charge profiles (Supplementary Fig. 6). The cycling performance of the Ca_x_Snǀǀ14PAQ cells at two different specific currents (260 and 130 mA g^−1^) is presented in Fig. 2d, delivering a reversible capacity of 152 mAh g^−1^ (corresponding a capacity retention of 61%) over 1200 cycles at 130 mA g^−1^ (The voltage profiles are shown in Supplementary Fig. 7). The cells could be cycled for more than 5000 cycles at 260 mA g^−1^ with a reversible capacity of 78 mAh g^−1^. The relatively fast capacity reduction in the first 100 cycles could be partly caused by the relatively large volume variation of Ca_2_Sn (~184%^29^) during the initial dealloying processes. It is worth noting that the degradation of the same 14PAQ cathode used for Mg battery systems has been observed in our previous investigation^36^, which could also account for a portion of the capacity fade of the Ca_x_Sn cells. Interestingly, after around 200 cycles, the cells exhibited a relatively stable capacity retention with a coulombic efficiency >99%, indicating highly reversible electrochemical processes at both cathode and anode sides.

**Fig. 3:**
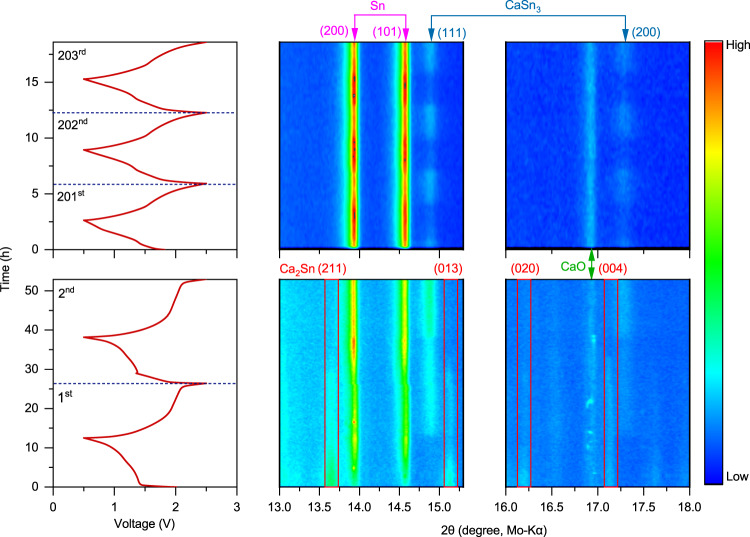
Phase analysis of the Ca–Sn alloy anodes during the electrochemical processes by in-situ XRD measurements. Isoplots of the in-situ XRD scans taken from the anode side of the Ca_x_Snǀǀ14PAQ cells during the first, second and from 201st to 203rd discharge/charge cycles with a specific current of 26 mA g^−1^ at 25 °C. The identity of each peak is shown along the top and the cell voltage profiles are shown on the left. ICSD-ID: Ca_2_Sn 659611, β-Sn 106072, CaSn_3_ 58934, Ca_36_Sn_23_ 54619 and CaO 26959.

We further investigated the morphological and compositional evolution of the alloy anode upon repeated calciation/decalciation in order to gain a deeper understanding of the properties of the Ca–Sn alloys. Insightful information about crystallographic and microstructural features of the Ca–Sn alloys at various electrochemical states have been unveiled by means of XRD and microscopic analysis. The phase evolution of the Ca_x_Sn anodes during the electrochemical processes was monitored by in-situ XRD with the Ca_x_Snǀǀ14PAQ cells operated in a voltage window of 0.5–2.5 V at a specific current of 26 mA g^−1^. Figure 3 displays the discharge/charge profiles of the 14PAQ cathode and the isoplots of the corresponding successive XRD scans of the alloy anode taken in the simultaneous decalciation/calciation processes in the selected cycles. Freshly assembled in-situ XRD cells showed an initial open circuit voltage (OCV) of ~1.9 V. The diffractograms of the Ca_x_Sn anode at the OCV-state resemble those of the as-prepared alloy powder (Supplementary Fig. 8). During the first discharge, a decrease in Ca_2_Sn peak intensity was observed immediately upon the onset of decalciation. While the Ca_2_Sn peaks continued to diminish in intensity, signals of ß–Sn phase began to appear and gradually became more intense, indicating that Ca_x_Sn was effectively dealloyed into β-Sn when discharging the cell. During charging, the enhancement of the Ca_2_Sn peaks was not observed, whereas new peaks for CaSn_3_ were identified. In the second cycle, the signal intensities of both Sn and CaSn_3_ further enhanced, and Ca_2_Sn phase was no longer detected, indicating that the formation of CaSn_3_ was kinetically more favorable than that of the Ca_x_Sn alloys in the reverse calcination process. (Ca_2_Sn is one of the most thermodynamically stable alloys among the Ca–Sn intermetallic compounds based on their formation enthalpies^37^). Further, in-situ XRD measurements conducted for the cell after 200 cycles clearly revealed the reversible decalciation/calciation of CaSn_3_ during repeated discharge and charge (Fig. 3). Calcium oxide (CaO) was also detected, which most likely formed during the in-situ measurements.

**Fig. 4:**
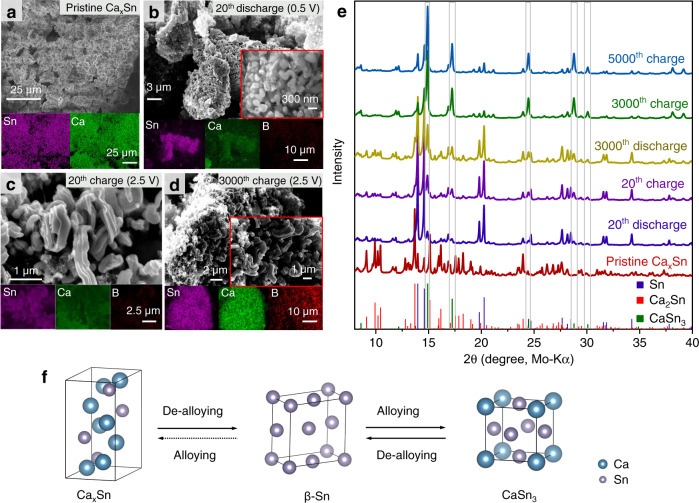
Microstructural and crystallographic analysis of the Ca–Sn alloy anodes at different electrochemical states. SEM images and elemental maps of the Ca_x_Sn anode material at the state of **a** pristine, **b** after 20th discharge to 0.5 V, **c** after 20th charge to 2.5 V, **d** after 3000th charge to 2.5 V. **e** XRD patterns of the Ca_x_Sn alloy from various discharge and charge states. **f** Scheme of the proposed phase evolution of the Ca_x_Sn anode during the electrochemical processes.

The SEM images shown in Fig. 4a–d indicate that the as-prepared pristine Ca_x_Sn aggregates have a typical particle size in the range 1–30 μm. After discharge and charge in the initial cycles, the alloy particles exhibited porous microstructure, e.g. as shown in the images for the samples from the 20th cycle. The image for the sample from the 3000th cycle shows that uniform rod-shaped crystalline solids (100-300 nm in width) appeared upon prolonged cycling. In addition, increased boron (B) containing species were detected with energy-dispersive X-ray spectroscopy (EDX), implying the decomposition of the electrolyte over 3000 cycles (Fig. 4d). The detailed EDX spectra and elemental maps are shown in Supplementary Fig. 9. The compositional changes after cycling were further tracked by ex-situ XRD (Fig. 4e), showing the presence of Ca_x_Sn was not detectable in the 20th cycle and the intensity of the Bragg peaks at 14.9° and 17.2° assigned for CaSn_3_ varied at the discharge and charge states. (The remaining Sn should originate from the decalciation of the excessive amount of the Ca_x_Sn anode material used for the cell construction). Interestingly, XRD diffraction patterns for the samples from the 3000th cycle imply that the redox reaction at the anode side mainly depended on the reversible alloying/dealloying of the CaSn_3_ phase. Based on these results, we propose the phase evolution and reaction mechanism of the Ca_x_Sn alloy anode as depicted in Fig. 4f, suggesting that the decalciation of the bulk Ca_x_Sn active material is a kinetically favorable process while the reverse process is largely restrictive; and the in-situ formed Sn preferably form CaSn_3_ in the subsequent recalciation process, which is most likely due to its the low volume change (7.3%^29^) during dealloying and alloying. Furthermore, dealloying Ca_x_Sn and CaSn_3_ might simultaneously occur during discharge until the active Ca_x_Sn in the anode completely transformed to Sn. These results indicate that the Ca_x_Sn alloy can undergo highly reversible redox reactions and may possess unique structural flexibility enabling circumvention of the structural stress during the electrochemical processes, which render them as promising candidates for use as anodes in Ca-ion batteries.

**Fig. 5:**
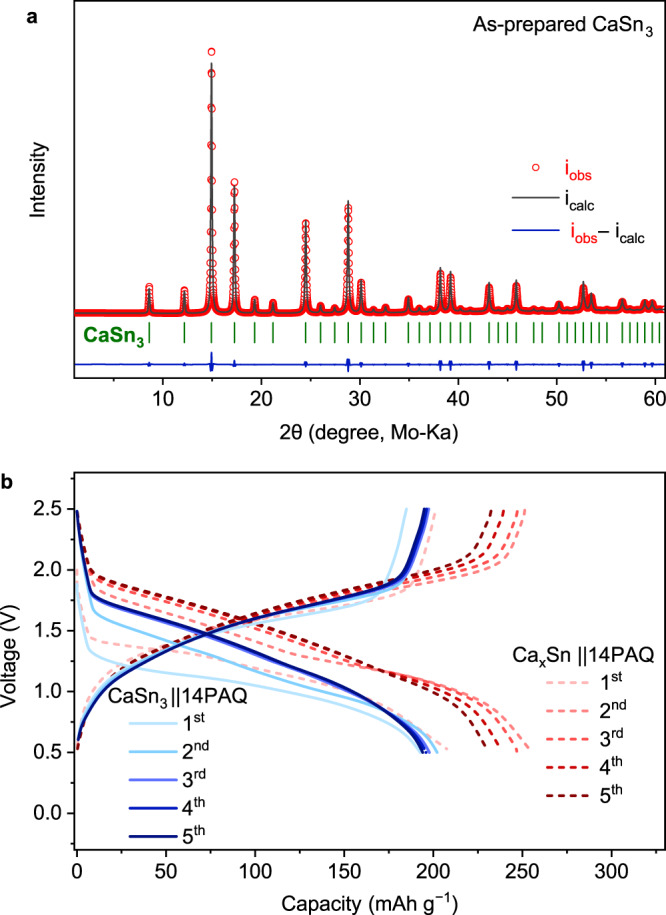
Crystallographic and electrochemical characterization of the CaSn_3_ alloy. **a** Rietveld refinement of the XRD pattern of as-synthesized CaSn_3_. The experimental diffraction pattern (red dots), calculated patterns (black lines), the difference curve (blue line) and Bragg diffraction positions for CaSn_3_ are presented. A single phase of CaSn_3_ can be identified (*χ*2 = 0.291, Rwp = 5.02%). **b** Galvanostatic discharge/charge profiles of CaSn_3_ǀǀ14PAQ cell in comparison with Ca_x_Snǀǀ14PAQ cell.

**Fig. 6:**
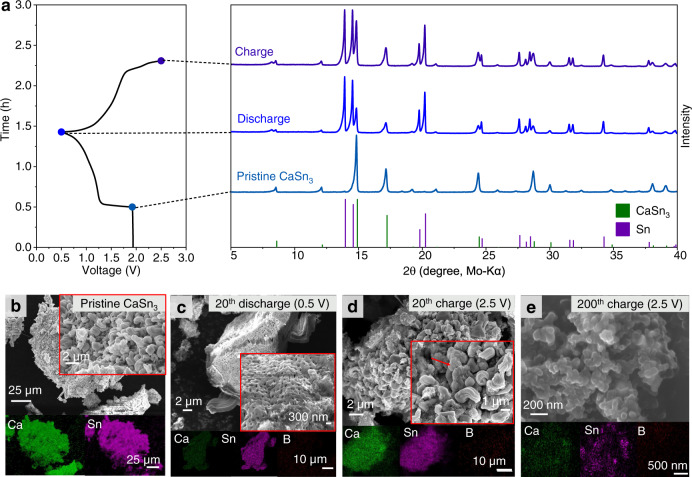
Phase and morphologic analysis of the CaSn_3_ anodes during the electrochemical processes. **a** Discharge/charge curves of CaSn_3_ǀǀ14PAQ cell during the first cycle with a specific current of 26 mA g^−1^ at 25 °C (left) and the ex-situ XRD patterns of the CaSn_3_ anodes at different electrochemical states (right). ICSD-ID: CaSn_3_ 58934, β-Sn 106072. SEM and EDX mapping images of **b** pristine CaSn_3_ alloy, **c** after 20th discharge to 0.5 V, **d** after 20th charge to 2.5 V, **e** after 200th charge to 2.5 V. The arrow indicates the round-shaped particles.

Intrigued by the electrochemical dealloying/alloying properties of the Ca_x_Sn alloy, we further examined the performance of CaSn_3_ with similar cell configuration, under same test conditions. Single CaSn_3_ phase was prepared by heating a mixture of Ca and Sn at a molar ratio of 1.1:3. The XRD patterns indicate that the as-prepared CaSn_3_ material is a pure single phase with high crystallinity (Fig. 5a). The CaSn_3_ǀǀ14PAQ cells delivered about 200 mAh g^−1^ capacity at 260 mA g^−1^ (1 C) and exhibited a lower average cell voltage compared to those of the Ca_x_Snǀǀ14PAQ cells (Fig. 5b), which is in agreement with the relatively high thermodynamic decalciation potential of CaSn_3_^29^. The electrochemical dealloying/alloying processes of the CaSn_3_ were characterized with ex- and in-situ XRD measurements. Figure 6a displays the voltage profiles of the 14PAQ cathodes and the corresponding XRD patterns of the CaSn_3_ alloy anodes taken in the simultaneous decalciation/calciation processes in the initial cycles. (The discharge/charge profiles for the sample preparation are shown in Supplementary Fig. 10) After discharging the cell to 0.5 V, a significant decrease in CaSn_3_ peak intensity was observed while the signals of ß-Sn became more intense, indicating that CaSn_3_ was effectively dealloyed when discharging the cell. After charging to 2.5 V, the CaSn_3_ peaks were enhanced, indicating the reversible alloying process. (Rietveld refinement XRD patterns of the discharged and charge samples and the corresponding compositional variations are provided in Supplementary Fig. 10 and Supplementary Tables 1 and 2.) The remained Sn after recharging might be ascribed to the excessive amount of the CaSn_3_ anode material used for the cell assembly and the limited calciation process with the micrometer-sized CaSn_3_. In addition, in-situ XRD results indicate similar trend of the phase variation of CaSn_3_ during discharge and charge, respectively (Supplementary Fig. 11). The microstructural changes of the CaSn_3_ during the electrochemical processes were further monitored by ex-situ SEM analysis. (The detailed EDX spectra and elemental maps are shown in Supplementary Fig. 12) Fig. 6b shows that the as-prepared bulk CaSn_3_ material consists of aggregates of uniform rod-like crystals with a length of a few micrometers and an average diameter of around 1 µm. The SEM images of the discharged sample from the 20th cycle revealed that the evenly distributed pores appeared over the alloy particles upon extraction of Ca (Fig. 6c). Some rounded micrometer-sized particles were observed in the charged sample as indicated by the arrow in Fig. 6d, which might be related to the remained Sn phase. Further, upon long cycling, both of the particle size and crystallinity were significantly reduced (Fig. 6e), implying the pulverization of the Sn phase formed during the repeated electrochemical dealloying/alloying CaSn_3_.

**Fig. 7:**
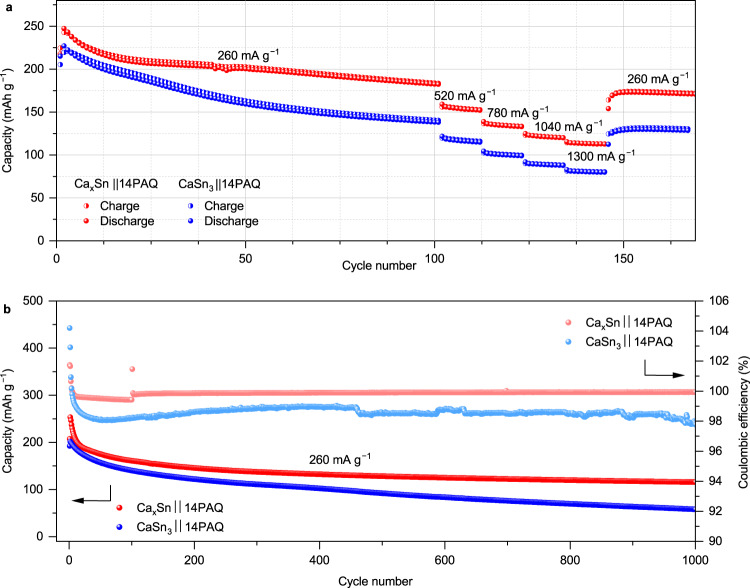
Electrochemical performance of the Ca–Sn alloy anode based batteries at 25 °C. **a** Rate performance and **b** cycling performance of CaSn_3_ǀǀ14PAQ and Ca_x_Snǀǀ14PAQ cells.

Owing to the low volume change during dealloying CaSn_3_, electrochemical kinetics might be expected. Nevertheless, the microstructure of the formed Sn and alloy phase may also play a vital role for the electrochemical performance. As discussed above, Ca_x_Sn alloy may undergo electrochemical phase transformation, forming new active nanosized CaSn_3_ phase. For a rate-performance comparison between the Ca_x_Snǀǀ14PAQ and CaSn_3_ǀǀ14PAQ battery systems, the cells were firstly operated at 260 mA g^−1^ (1 C) for 100 cycles, respectively. As presented in Fig. 7a, the cell starting with Ca_x_Sn exhibited higher capacities at different current densities while the CaSn_3_ cell showed relatively faster capacity fade in the initial 100 cycles at 260 mA g^−1^ and less capacities at the corresponding current rates. (The voltage profiles at various current densities are presented in Supplementary Fig. 13). Further, compared to the Ca_x_Snǀǀ14PAQ cells, the CaSn_3_ǀǀ14PAQ cells exhibited an inferior capacity retention and coulombic efficiency during cycling at 260 mA g^−1^ for 1000 cycles as displayed in Fig. 7b. This could be ascribed to the low electrochemically active surface area of the Sn phase formed from the bulk CaSn_3_ and possible pulverization during cycling as shown in Fig. 6b–e. These results suggest that the different microstructure of the in-situ formed crystalline Sn is strongly related to the starting bulk Ca–Sn alloy and the coral-like interconnected 3D structure of the Sn phase formed during electrochemical decalciation of the Ca_x_Sn as shown in Fig. 4b may be crucial for the better cycling stability. Similar self-transformation phenomena of a Sn anode and its beneficial effect on the stable cycling performance have also been discovered in Na-ion systems^38^. In addition, the specific capacity of both alloys was estimated by pairing with a cathode with a higher active polymer loading, respectively, where a lower capacity (62 mAh g^−1^) for CaSn_3_ was estimated compared to that of Ca_x_Sn (109 mAh g^−1^) (Supplementary Fig. 14). As the Ca–Sn alloys containing higher Ca content potentially provide higher capacity, dedicated investigations of Ca-rich Sn alloys will be conducted in future work. The results presented in this work demonstrate the feasibility of Ca–Sn alloys for use as an anode material in Ca-based chemistries, which may offer a new approach for developing viable Ca batteries. Supplementary Table 3 presents a performance comparison of the state-of-art multivalent metal batteries with single metal-ion storage mechanisms.

In summary, we have developed a Ca full cell comprised of a quinone based polymer cathode and a Ca–Sn alloy anode in combination with the efficient borate Ca[B(hfip)_4_]_2_ electrolyte. This full cell prototype exhibited a cell voltage of about 1.8 V and could be operated for at least 5000 cycles at a specific current of 260 mA g^−1^ (1 C). Electron microscopic and crystallographic investigations on the compositional and microstructural features of the alloy electrodes during the electrochemical processes unveiled that micrometer-sized bulk Ca–Sn electrode materials could be transformed to active Sn through the initial electrochemical dealloying and the in-situ formed unique interconnected porous structure is beneficial for the cycling stability. This study shows that microstructure, and not only alloy composition, are critical considerations for the reversibility of alloy anodes for Ca batteries. In addition, micrometer-sized Ca–Sn alloy powder can be straightforwardly synthesized, which could be considered as a promising approach for large-scale applications. The usage of a full-cell configuration instead of the conventional half-cell setup may provide a feasible option for discovering new electrode materials for Ca-ion batteries. Future work on tailoring alloy compositions and exploring high-voltage cathodes could result in energy-denser full batteries.

## Methods

### Materials preparation

#### Ca–Sn alloys

The Ca–Sn alloys were prepared by mixing calcium granules (99.5%, Alfa Aesar, 16 mesh) and tin powder (99.8%, Alfa Aesar, 325 mesh) at a molar ratio of 2.2:1 and 1.1:3 for Ca_x_Sn, CaSn_3_ alloy, respectively. The mixture of Ca (1.15 g, 28.69 mmol) and Sn (1.52 g, 12.80 mmol) for Ca_x_Sn or Ca (1.00 g, 24.95 mmol) and Sn (8.08 g, 68.05 mmol) for CaSn_3_ was loaded into a zirconium oxide crucible and put into a quartz tube under argon, then heated up to 900 °C (at a heating rate of 100 °C h^−1^) and dwelled for 1 h. After cooling down to the ambient temperature, the alloy products were collected and grounded for 30 min with an agate mortar prior to use.

#### Ca[B(hfip)_4_]_2_ electrolyte

The calcium tetrakis(hexafluoroisopropyloxy)borate Ca[B(hfip)_4_]_2_·4DME salt and the electrolyte solutions were synthesized according to literature^21^. Typically, calcium borohydride Ca(BH_4_)_2_∙2THF (3.21 g, 15.0 mmol) was dissolved into 60 mL DME in a two necked Schlenk flask equipped with a reflux condenser. Hexafluoroisopropanol (CF_3_)_2_CHOH (12.8 ml, 122 mmol) were added dropwise into the stirred solution of Ca(BH_4_)_2_. The reaction mixture was refluxed for 4 h. Then the solvent was removed by vacuum. The solid raw product was dried at 40 °C overnight followed by 60 °C for 24 h, yielding 21.8 g (83%) of final product. 0.25 M electrolyte was prepared by dissolving Ca[B(hfip)_4_]_2_∙4DME in a proper amount of DME with a volumetric flask.

#### 14PAQ cathode material

14PAQ was prepared according to the procedures reported in literature^39^. Typically, bis(1,5-cyclooctadiene)nickel(0) (Ni(COD)_2_, 2.2 g, 8 mmol), 2,2'-bipyridine (1.25 g, 8 mmol), and 1,5-cyclooctadiene (COD, 0.74 ml, 6 mmol) were first dissolved in 60 mL dimethylformamide (DMF) in a Schlenk flask. 1,4-dichloroanthraquinone (1,4-DCAQ, 2.2 g, 8 mmol) was dissolved in 40 ml DMF and added into the above solution. These procedures were carried out in an argon-filled glove box (O_2_, H_2_O < 0.1 ppm). Subsequently, the polymerization reaction was carried out by heating the mixed solution at 60 °C for 48 h under argon using a Schlenk line in fume hood. Then the reaction mixture was poured into a beaker containing 150 ml, 0.5 M hydrochloric acid solution. The yellow product precipitated immediately. Then it was filtered and washed with DMF, 0.5 M hydrochloric acid, warm deionized water, and methanol for several times. Subsequently, it was purified by re-precipitation by dissolving the raw product in chloroform and adding methanol afterwards. Finally, the polymer material was further washed through Soxhlet extraction in DME for 48 h to remove the soluble short-chain polymer and dried at 85 °C for 12 h under vacuum.

The 14PAQ@KB composite was prepared by a solution dispersion method. 14PAQ was dissolved in chloroform and mixed with Ketjenblack EC600JD (KB) at a mass ratio of 1:1. After stirring under argon for 24 h, the suspension was treated by ultrasonic mixing for 2 h. After removal of chloroform by vacuum, the composite material was dried at 80 °C in vacuum for 3 h and then ball-milled under argon at 200 rpm for 2 h to obtain a fine powder.

### Materials characterization

The synthesized Ca[B(hfip)_4_]_2_∙4DME and 14PAQ was characterized by magnetic resonance spectroscopy (NMR) with a Bruker Advance II 500 spectrometer using THF-d_8_ and CDCl_3_ as solvent, respectively. Additionally, the 14PAQ cathodes at different electrochemical states were analyzed with Fourier-transform infrared spectroscopy (FT-IR, Spectrum Two, PERKIN ELMER) with the attenuated total reflection (ATR) in an Ar-filled glovebox. To prepare the 14PAQ cathode samples after discharge and charge process, the cells were disassembled in an Ar-filled glovebox and the residual electrolyte salt on the cathodes was washed with anhydrous DME for three times. Afterwards, the samples were dried in a glass tube by vacuum at the ambient temperature overnight in a fume hood.

The as-prepared Ca–Sn alloy powders and the alloy electrodes were characterized using a scanning electron microscope (SEM, ZEISS LEO 1530 at 15 kV) equipped with an energy-dispersive X-ray (EDX) detector. The X-Ray diffraction (XRD) measurements were performed by a STOE STADI P diffractometer with the Mo Kα radiation (*λ* = 0.709300 Å) operated at 50 kV and 40 mA. All the XRD patterns were recorded in a Bragg angle range of 1°‒63° at steps of 1° and a scan rate of 1° min^−1^. For the in-situ XRD measurements, the coin cell (CR2032) with a glass window in a diameter of 5 mm was assembled in an Ar-filled glove box, in which stainless steel mesh (type 316) was used as the current collectors for both electrodes. The cells were operated at 26 mA g^−1^ (0.1 C) while the XRD scans were taken from the anode side with transmission geometry in a Bragg angle range of 1°‒43° at steps of 1° and a scan rate of 1° min^−1^. The Rietveld refinement of the collected XRD patterns were conducted with Full Prof_suite programs.

To prepare the alloy anode samples after discharge and charge processes, the cells were disassembled in an Ar-filled glovebox and the residual electrolyte salt on the cathodes was washed with anhydrous DME for three times. Then the samples were dried in a glass tube by vacuum at the ambient temperature overnight in a fume hood. For SEM measurements, the alloy anode samples were transferred with an airtight sample holder. They were shortly exposed to ambient air when being put into the SEM chamber. For ex-situ XRD measurements, the alloy anode samples were sealed in a glass capillary in an Ar-filled glove box.

### Electrochemical measurements

All of the samples were handled in an argon-filled glove box (H_2_O, O_2_ < 0.1 ppm). The Ca–Sn alloy anodes were prepared by mixing the Ca–Sn alloy powders, KB and polytetrafluoroethylene (PTFE) binder at a mass ratio of 6:3:1 with an agate mortar. Then, the mixture containing 9 mg of the alloy was press on to a stainless steel mesh with a 10 mm die set using a hydraulic press machine, forming the alloy electrode with a thickness of about 100 µm and a mass loading of ~12 mg cm^−2^. The cathodes were prepared by mixing the 14PAQ/KB composite with PTFE at a mass ratio of 9:1 and a small amount of isopropanol; then the slurry was pasted onto an aluminum mesh at 80 °C and dried in vacuum at 85 °C for 12 h. The mass loading of the active 14PAQ for the cathodes was ~1.5 mg cm^−2^. The galvanostatic discharge/charge tests were conducted with an Arbin or BCS 805 battery tester (Bio-Logic) using coin cells (CR2032) comprising a Ca–Sn alloy anode, a 14PAQ@KB cathode, and Whatman GF/C separators soaked with 0.25 M Ca[B(hfip)_4_]_2_/DME electrolyte. The cutoff voltage of Caǀǀ14PAQ cell was 0.5 V for discharge and 3.0 V for charge. The cutoff voltage of Ca_x_Snǀǀ14PAQ and CaSn_3_ǀǀ14PAQ was 0.5 V for discharge and 2.5 V for charge, respectively. the Cyclic voltammetry (CV) was performed with a VMP3 potentiostat (Bio-Logic) using a PAT-Cell (EL-Cell) with the CaSn-alloy as both the counter and reference electrode, 14PAQ as the working electrode and Ca[B(hfip)_4_]_2_/DME electrolyte using at a scan rate of 0.1 mV min^−1^. All the electrochemical cells were kept in a BINDER climate chamber with a constant temperature of 25 °C for the measurements.

## Supplementary information

Supplementary Information

## Data Availability

The datasets generated during and/or analyzed during the current study are available from the corresponding author on reasonable request.
