# Author Correction: Calcium-tin alloys as anodes for rechargeable non-aqueous calcium-ion batteries at room temperature

**DOI:** 10.1038/s41467-022-33113-2

**Published:** 2022-09-07

**Authors:** Zhirong Zhao-Karger, Yanlei Xiu, Zhenyou Li, Adam Reupert, Thomas Smok, Maximilian Fichtner

**Affiliations:** 1 Helmholtz Institute Ulm (HIU) Electrochemical Energy Storage, Helmholtzstr. 11, D-89081 Ulm, Germany; 2 Institute of Nanotechnology, Karlsruhe Institute of Technology (KIT), P.O. Box 3640, D-76021 Karlsruhe, Germany

**Keywords:** Batteries, Energy storage, Nanoscale materials, Energy

Correction to: *Nature Communications* 10.1038/s41467-022-31261-z, published online 04 July 2022

The original version of this Article contained an error in the Methods, section “Ca–Sn alloys”, which incorrectly read ‘Sn (21.52 g, 12.80 mmol).’ The correct version replaces this with ‘Sn (1.52 g, 12.80 mmol).’ This has been corrected in both the PDF and HTML versions of the Article.

