# Supplementary material for: Calcium-tin alloys as anodes for rechargeable non-aqueous calcium-ion batteries at room temperature
Source: Nat Commun. 2022 Jul 4;13:3849. doi: 10.1038/s41467-022-31261-z (PMC9253317; doi:10.1038/s41467-022-31261-z)
Supplement: Supplementary file 1 — Supplementary Information [file 41467_2022_31261_MOESM1_ESM.pdf]

Supplementary Information

Calcium-tin alloys as anodes for rechargeable non-aqueous calcium-ion batteries at room temperature

Zhirong Zhao-Karger,*+[1,2] Yanlei Xiu,+[1] Zhenyou Li,[1] Adam Reupert,[1] Thomas Smok,[1] Maximilian Fichtner [1,2]

[1]*Helmholtz Institute Ulm (HIU) Electrochemical Energy Storage, Helmholtzstr. 11, D-89081, Ulm, Germany*

[2]*Institute of Nanotechnology, Karlsruhe Institute of Technology (KIT), P.O. Box 3640, D-76021 Karlsruhe, Germany*

[+] These authors contributed equally to this work.

Supplementary data

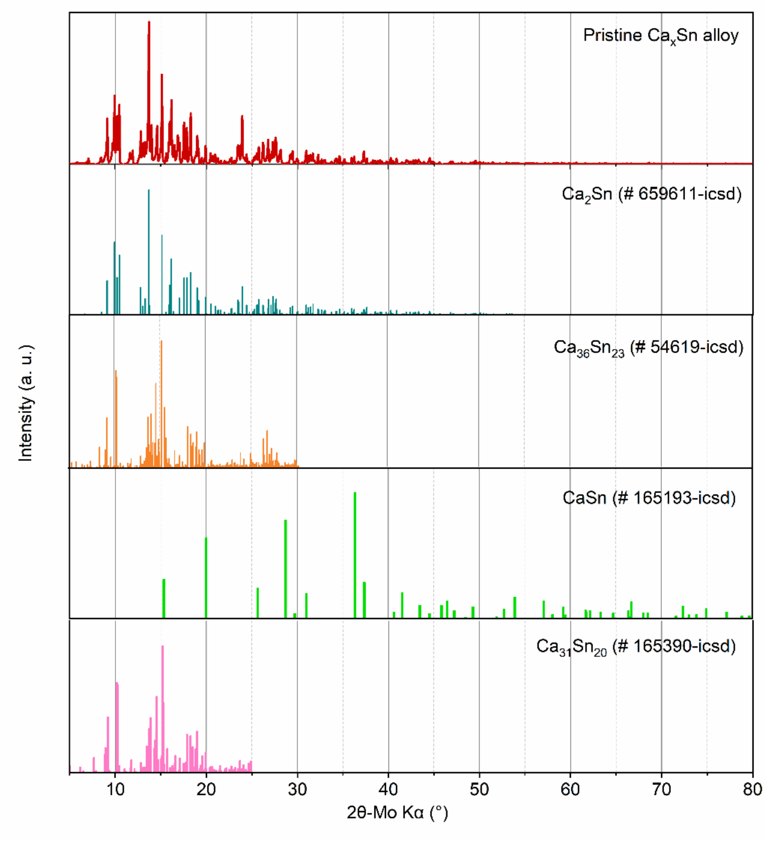

Supplementary Figure 1. Phase characterization of the Ca-Sn alloy. XRD patterns of the as-prepared Ca-Sn alloy and the related reference patterns.

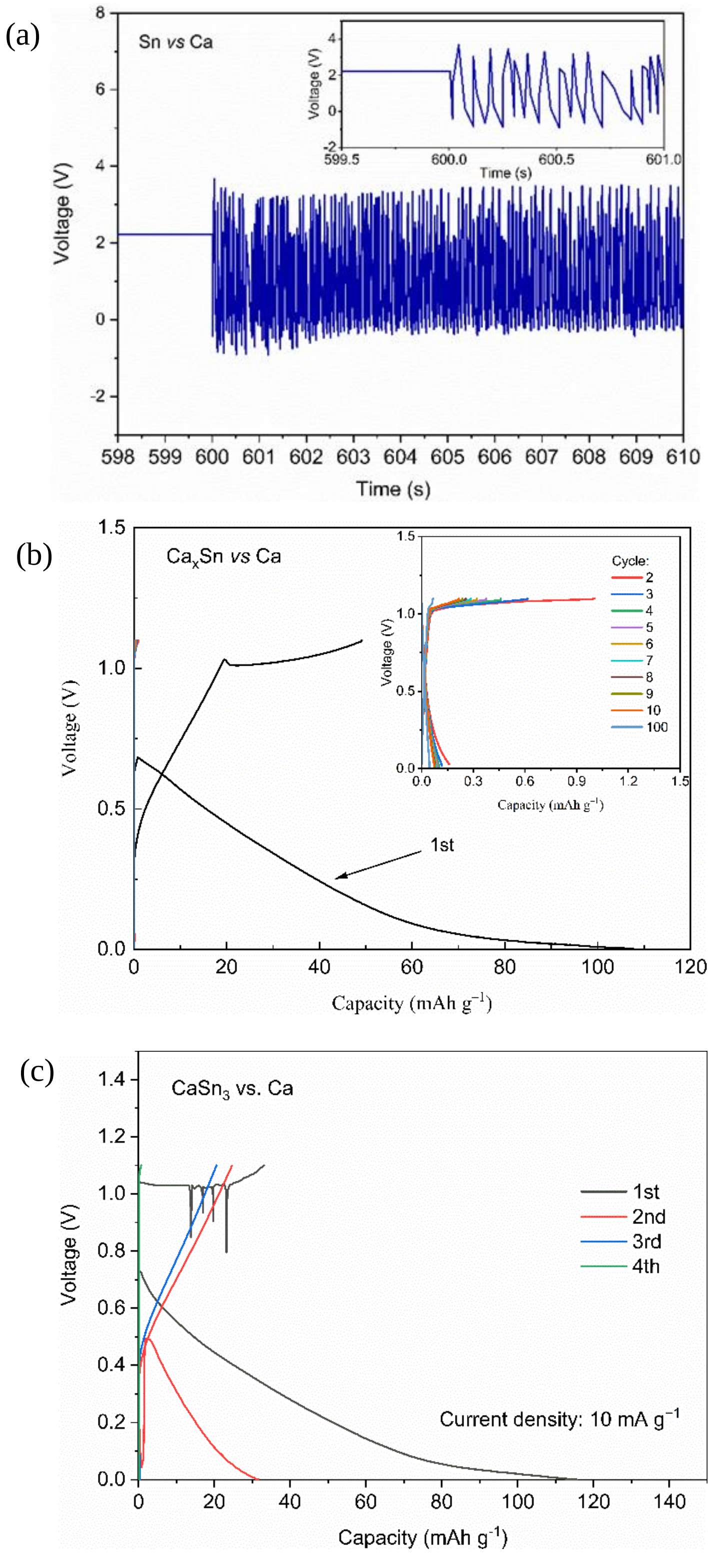

Supplementary Figure 2. Electrochemical measurements of Ca-based half-cells. Galvanostatic voltage profiles of the half-cells with a Ca counter electrode coupled with (a) Sn at a current density of 0.1 mA g^{-1}, (b) Ca_xSn electrode and (c) $CaSn_3$ electrode at a current density of 10 mA g^{-1}, respectively.

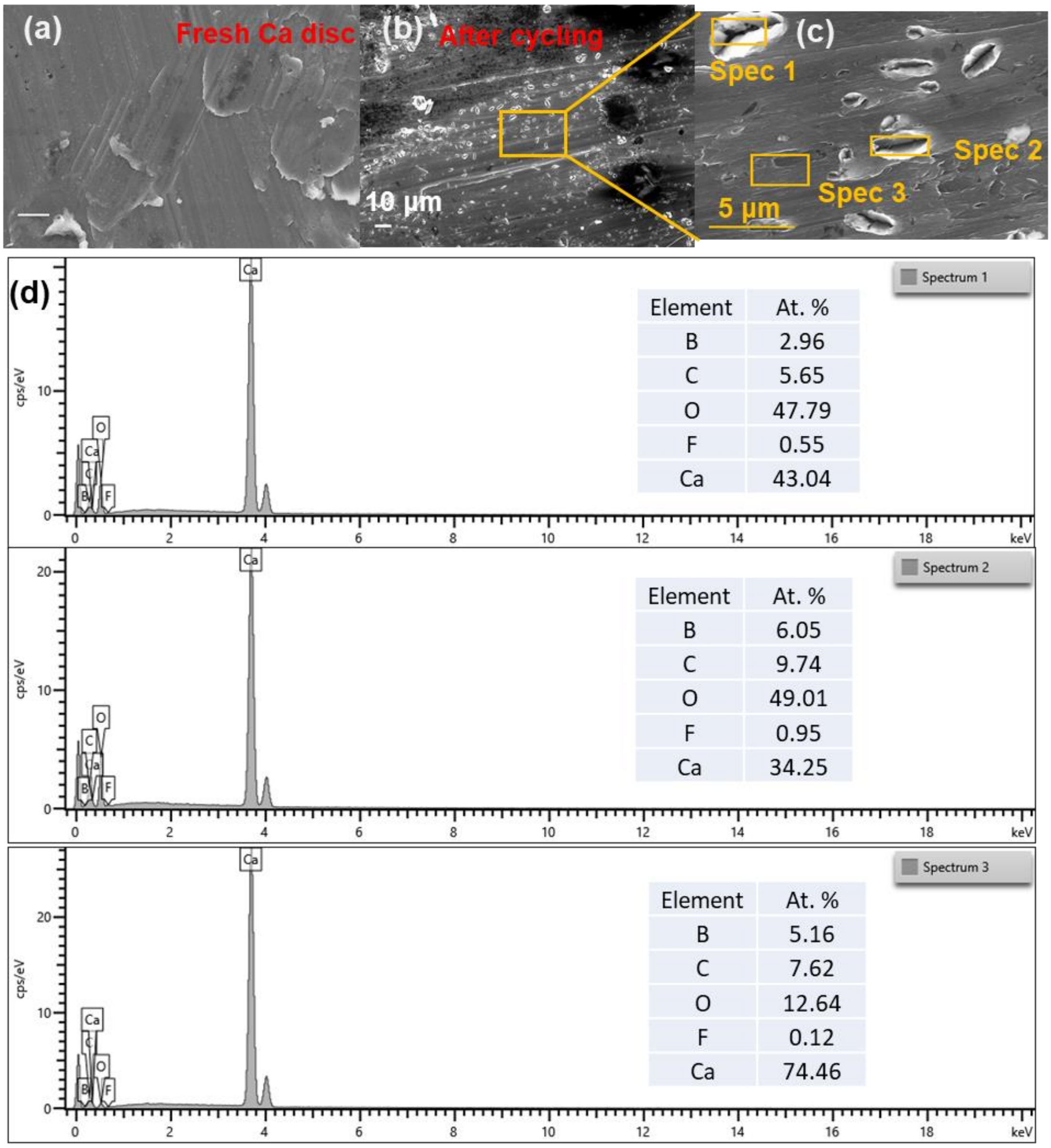

Supplementary Figure 3. SEM and EDX analysis of the Ca electrode. SEM images of the Ca counter electrode: (a) the initial Ca pellet, (b) after half-cell testing with the Ca_xSn electrode, (c) the area with crater-like spots and (d) the corresponding EDX spectra of the selected regions.

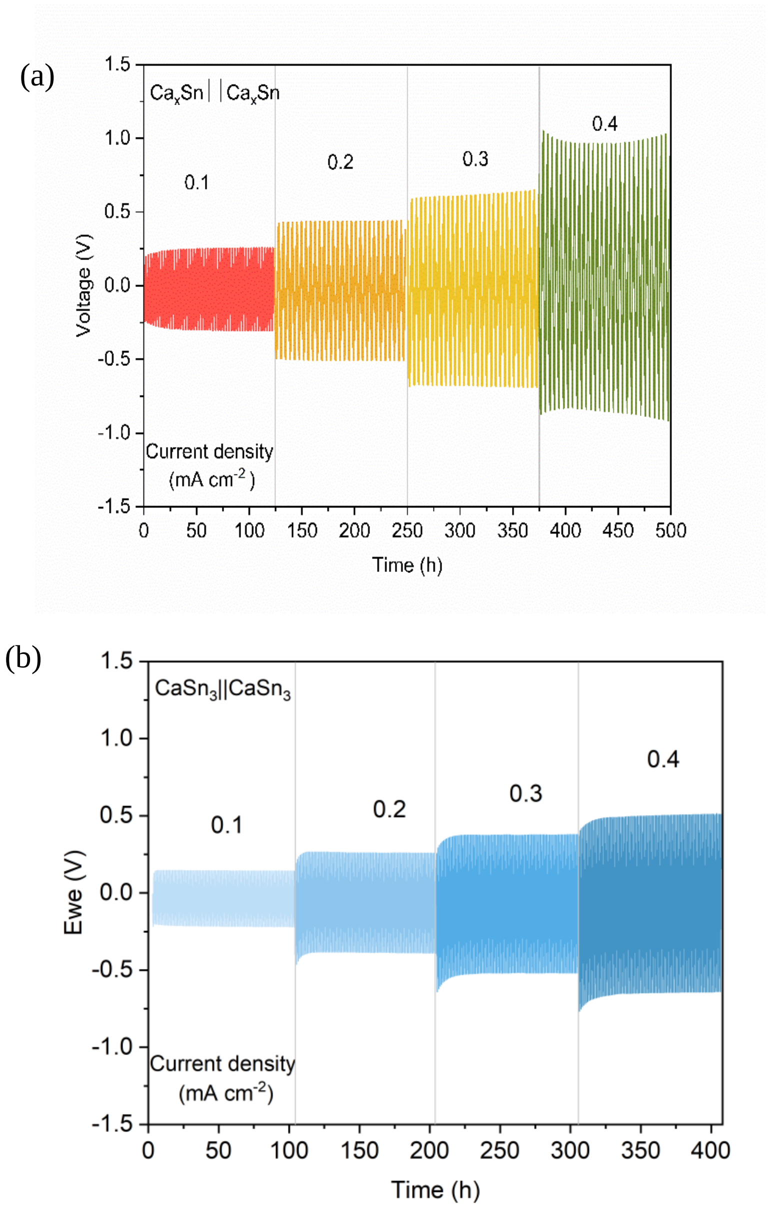

Supplementary Figure 4. Electrochemical characterization of the Ca-Sn alloys in symmetric cells. Voltage profiles of (a) the Ca_xSn alloy electrode and (b) the Ca_xSn alloy electrode in symmetric cells at different current densities.

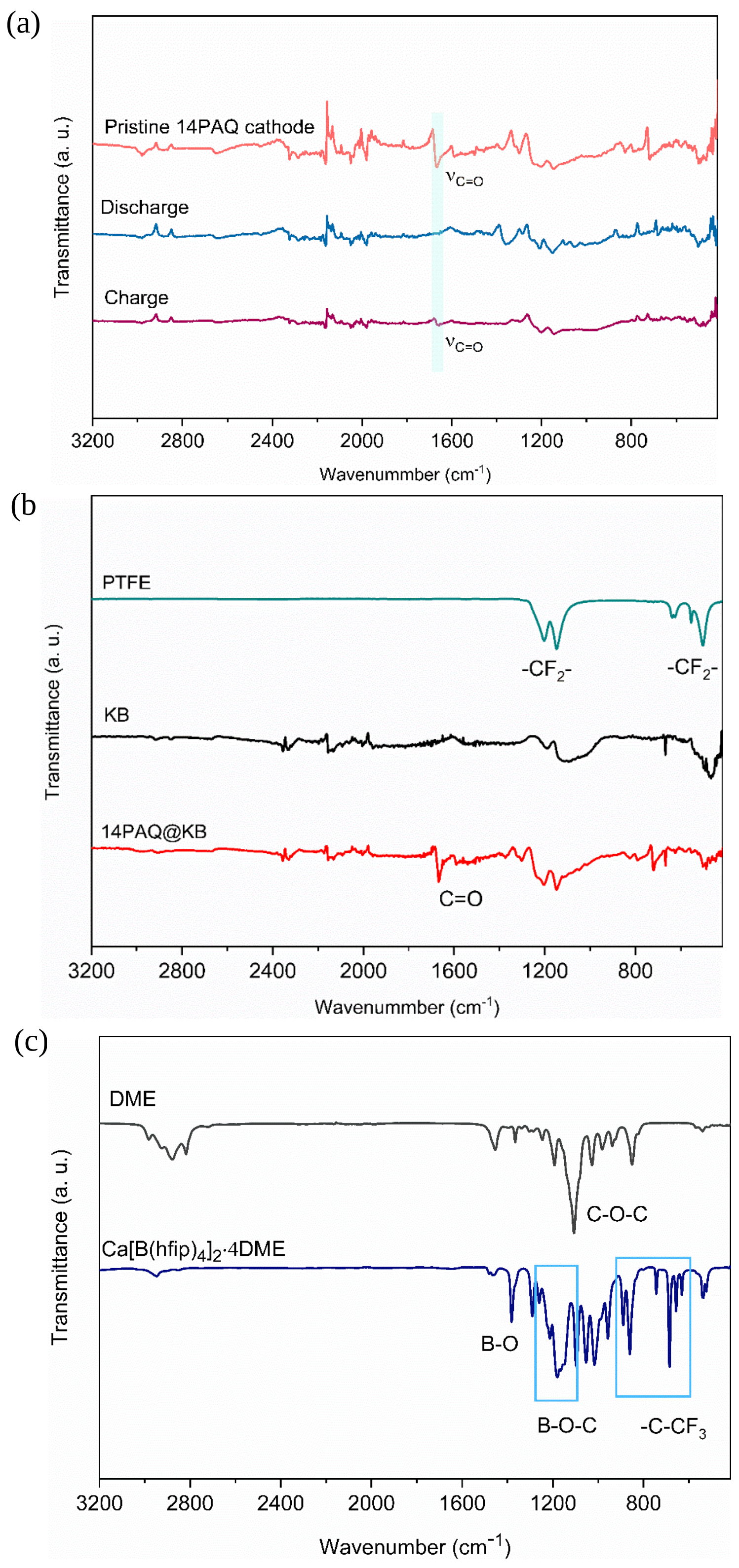

Supplementary Figure 5. Chemical analysis of the 14PAQ cathodes by IR. IR spectra of (a) 14PAQ@KB cathode at different electrochemical states, (b) KB and PTFE and (c) DME and Ca[B(hfip)$_4$]$_2$•4DME as references.

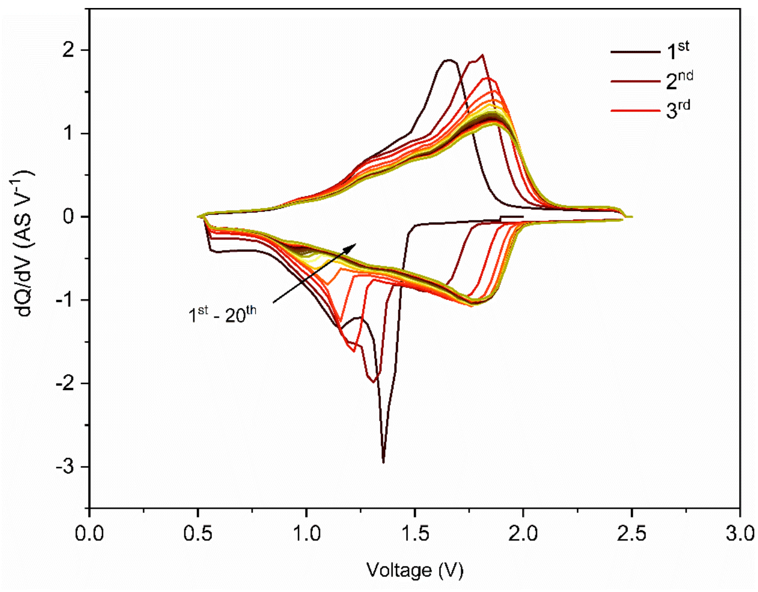

Supplementary Figure 6. Electrochemical characterization of $Ca_xSn||14PAQ$ cell.

Differential capacity analysis of the $Ca_xSn||14PAQ$ cell at 260 mA g^{-1} (1C).

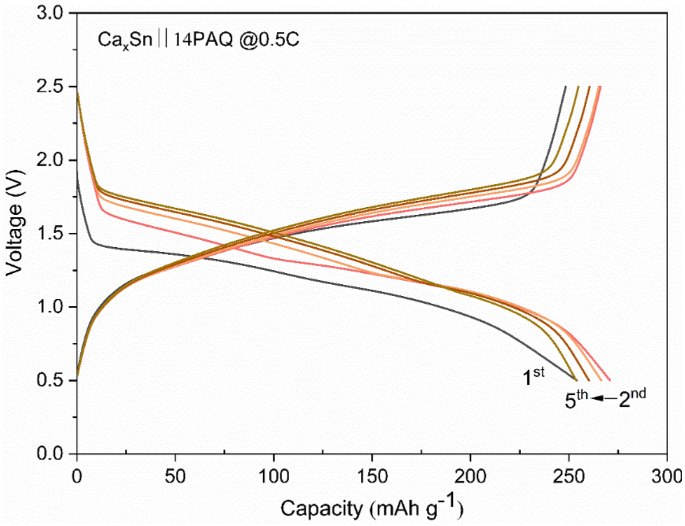

Supplementary Figure 7. Electrochemical characterization of $Ca_xSn||14PAQ$ cell.

Voltage profiles of the first 5 cycles of the $Ca_xSn||14PAQ$ cell at 130 mA g^{-1} (0.5C).

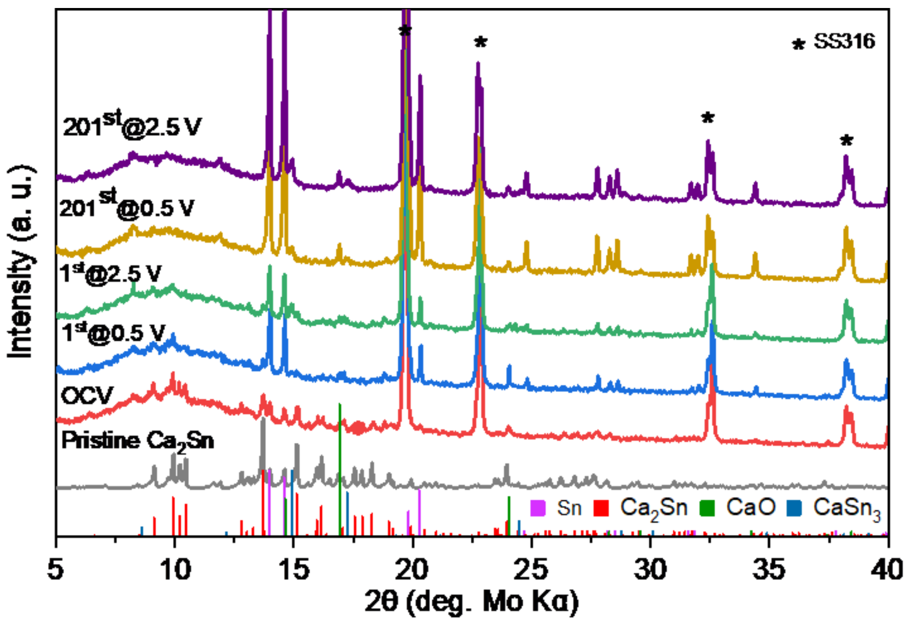

Supplementary Figure 8. Phase analysis of the Ca-Sn alloy anodes. XRD patterns of Ca_xSn-anode at various electrochemical states from the *in-situ* XRD scans of the Ca_xSn||14PAQ cell.

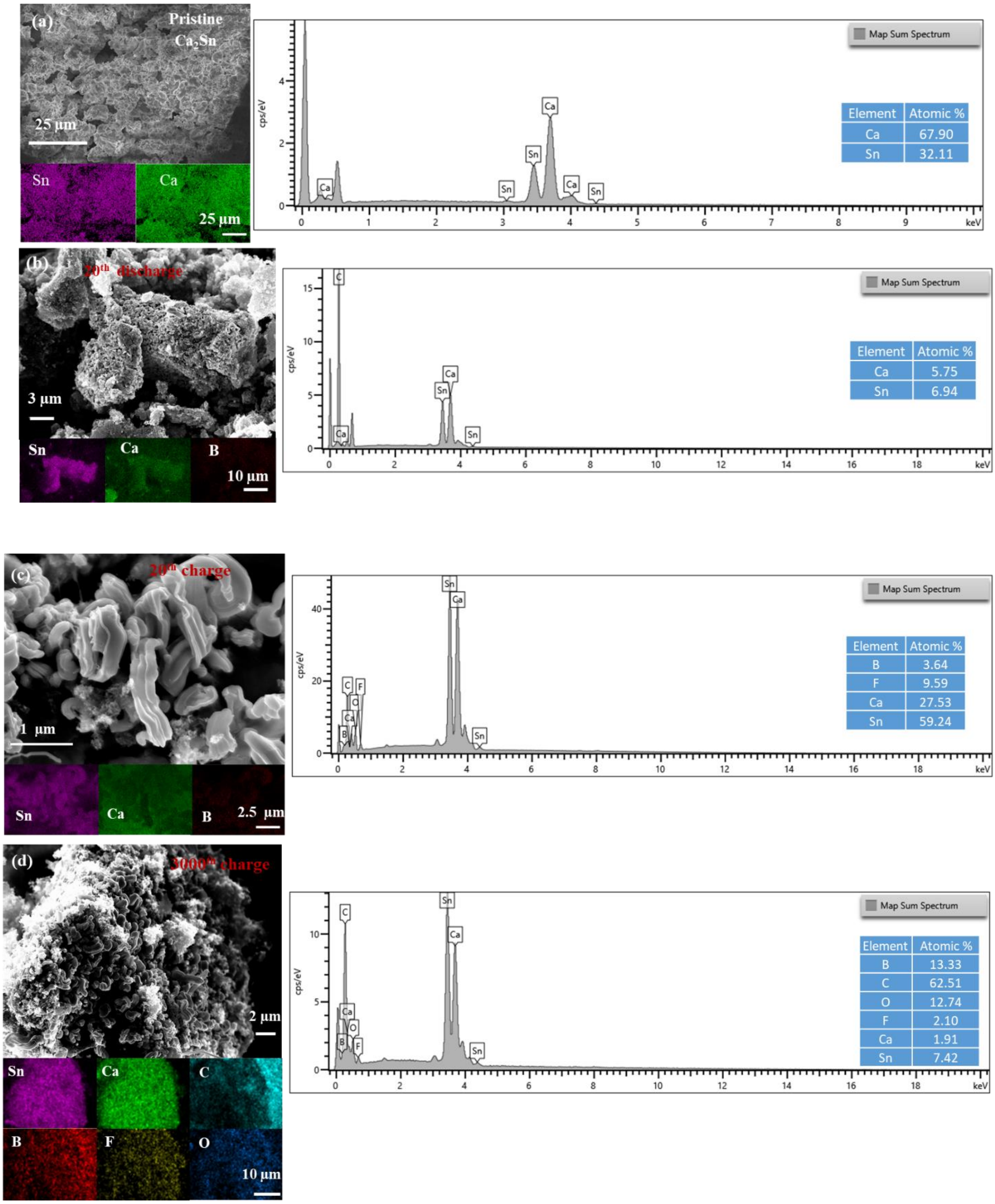

Supplementary Figure 9. SEM and EDX analysis of the Ca_xSn-anodes. SEM images and corresponding EDX spectra and elemental maps (Ca Kα1, Sn Lα1, C Kα1_2, B Kα1_2, F Kα1_2, O Kα1 lines) of the charged Ca_xSn-anode at different electrochemical states.

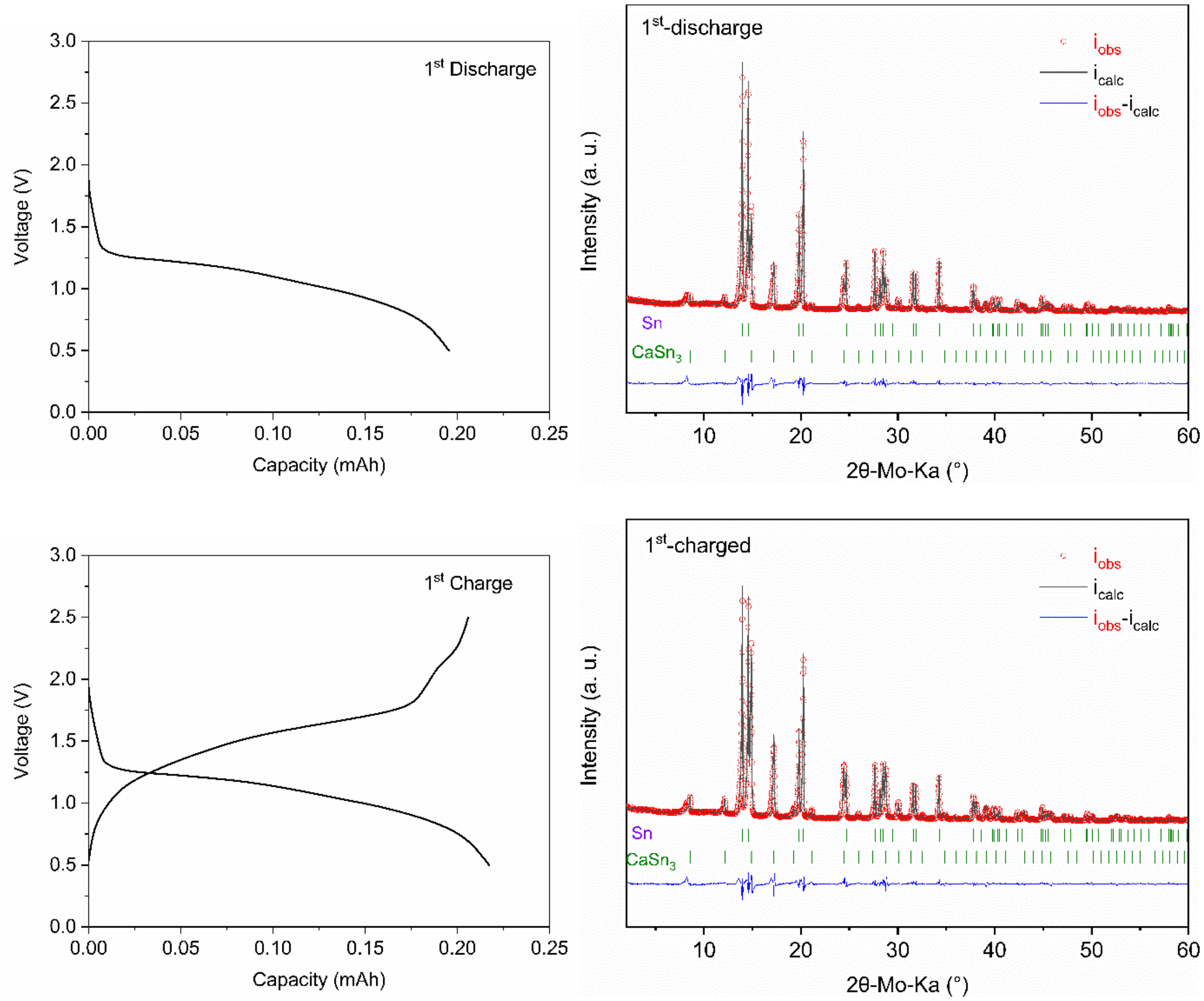

Supplementary Figure 10. Electrochemical and crystallographic characterizations of the $CaSn_3$ alloy anodes. Discharge/charge profiles of the $CaSn_3$||14PAQ cells at 1C (left) and the corresponding Rietveld refinement XRD patterns for $CaSn_3$ anode after discharge and charged, respectively (Right). The experimental diffraction pattern (red dots), calculated patterns (black lines), the difference curve (blue line) and Bragg diffraction positions for Sn and $CaSn_3$ are presented in Supplementary Table 1.

Supplementary Table 1. The corresponding parameters in Rietveld analysis of the $CaSn_3$ anode.

1st Cycle	Discharge		Charge	
Phase	Bragg R-factor (%)	Phase ratio (mol %)	Bragg R-factor (%)	Phase ratio (mol %)
Sn	2.88	90.7	2.50	83. 5
$CaSn_3$	8.25	9.3	6.41	16.5
Refinement parameter	$\chi 2$ = 9.97	Rwp = 15.9%	$\chi 2$ = 8.58	Rwp = 15.9%

The mass loading of the $CaSn_3$ alloy in the anode is ~7mg (0.0177 mmol, $CaSn_3$), in which the Sn-element is 0.0531 mmol and assumed constant for a further estimation. Based on the phase composition from the Rietveld refinement, the content variation of Sn and $CaSn_3$ is presented in Supplementary Table 2. The specific capacity of $CaSn_3$ is estimated as approximately 145.69 mAh g^{-1} corresponding to the change of $CaSn_3$ (1.51 mg) and a released capacity (0.22 mAh).

Supplementary Table 2. Phase composition of the $CaSn_3$ anode at different states in the 1st cycle.

1st Cycle	Charge (mg)	Discharge (mg)
Sn	5.26	5.72
$CaSn_3$	3.47	1.96

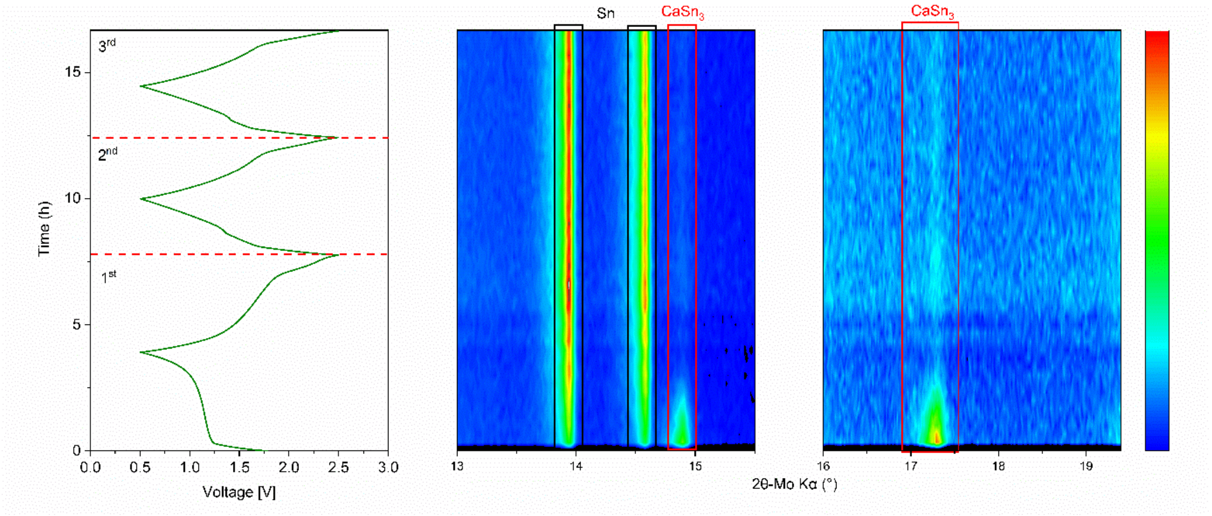

Supplementary Figure 11. Phase analysis of $CaSn_3$ anode by in-situ XRD measurements. Isoplots of the in-situ XRD scans taken from the anode side of the $CaSn_3$||14PAQ cells during the first, second discharge/charge cycle at 26 mA g^{-1}, respectively.

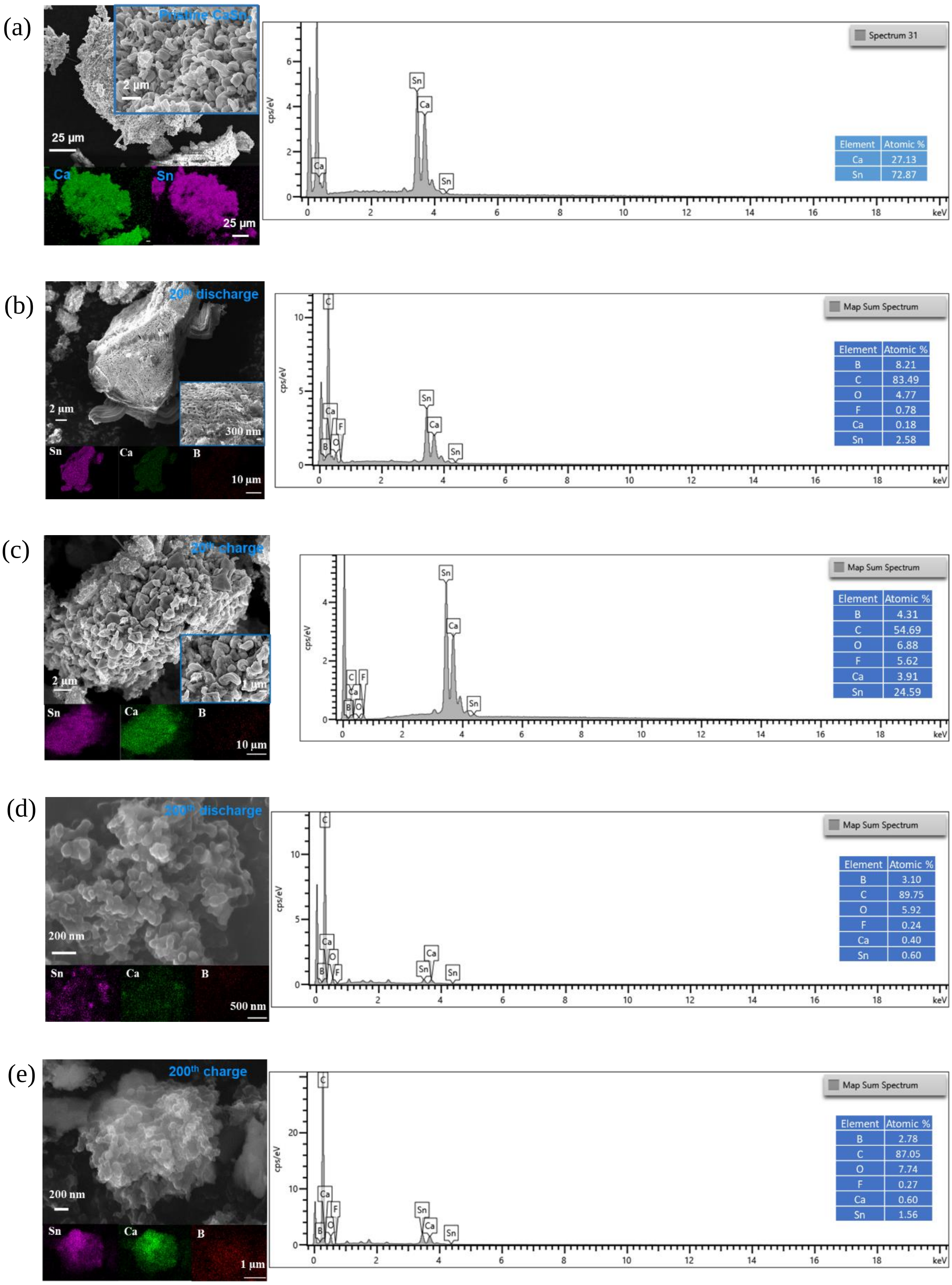

Supplementary Figure 12. SEM and EDX analysis of the $CaSn_3$ anodes. SEM images and corresponding EDX spectra and elemental maps (Ca Kα1, Sn Lα1, C Kα1_2, B Kα1_2, F Kα1_2, O Kα1 lines) of the $CaSn_3$-anode at different electrochemical states.

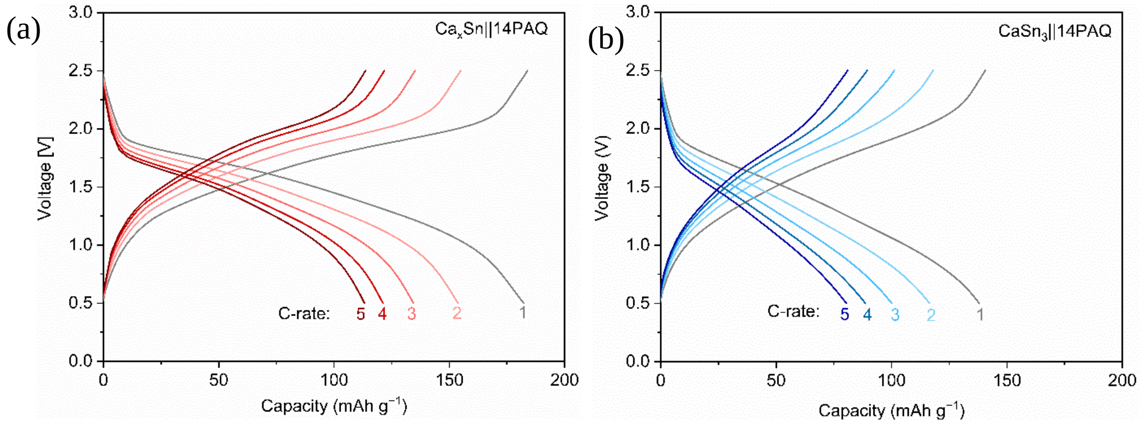

Supplementary Figure 13. Electrochemical performance of the Ca-Sn alloy based cells. Voltage profiles of (a) Ca_xSn||14PAQ in the 100th cycle at 260 mA g^{-1}, 101th cycle at 520 mA g^{-1}, 102th cycle at 1040 mA g^{-1} and 103th cycle at 1300 mA g^{-1}, (b) $CaSn_3$||14PAQ cell in the 5th cycle at 260 mA g^{-1}, 6th cycle at 520 mA g^{-1}, 7th cycle at 1040 mA g^{-1} and 8th cycle at 1300 mA g^{-1}.

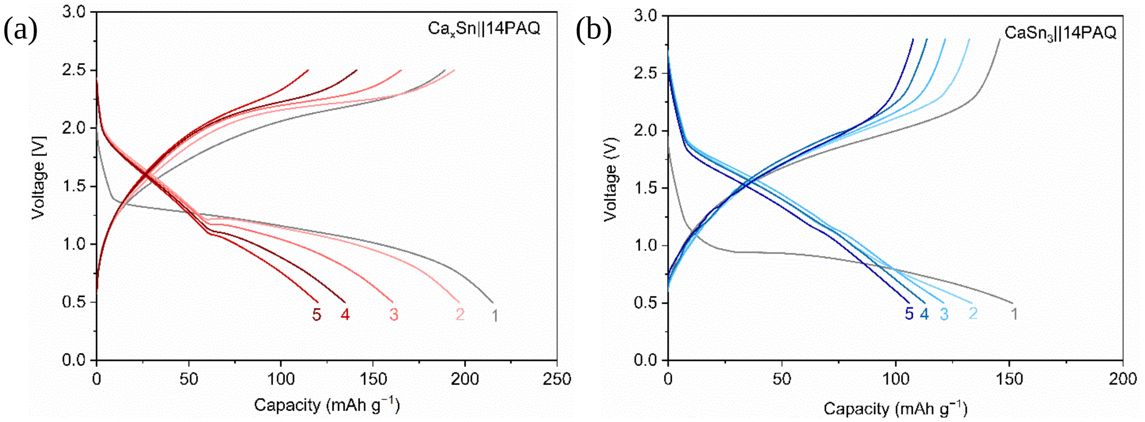

Supplementary Figure 14. Electrochemical performance of the Ca-Sn alloy based cells. (a) Voltage profiles of the Ca$_x$Sn||14PAQ cell with a higher active cathode material loading at 26 mA g^{-1} for the estimation of the specific capacity of the Ca$_x$Sn alloy. (b) Voltage profiles of the CaSn$_3$ cell with a higher active cathode material loading at 26 mA g^{-1} for the estimation of the specific capacity of the CaSn$_3$ alloy

The anodes and cathodes for both cells were prepare with a diameter of 1 cm with an area of 0.785 cm^2. For Ca$_x$Sn||14PAQ cell, the mass loading of the active material 14PAQ in the cathode was 4.7 mg, corresponding to 6.11 mg cm^{-2}. The mass loading of the active material Ca$_x$Sn in the anode was 9.5 mg, corresponding to 12.1 mg cm^{-2}, Ca$_x$Sn:14PAQ = 2:1 (mass ratio). Specific capacity of Ca$_x$Sn was estimated to be approximately 109 mAh g^{-1} based on the highest capacity 215 mAh g^{-1}.

For CaSn$_3$||14PAQ cell, the mass loading of the active material 14PAQ in the cathode was 5.7 mg, corresponding to 7.26 mg cm^{-2}. The mass loading of the active material Ca$_x$Sn in the anode was 13.8 mg, corresponding to 17.6 mg cm^{-2}, CaSn$_3$:14PAQ = 2.4:1 (mass ratio). Specific capacity of CaSn$_3$ was estimated to be approximately 62 mAh g^{-1} based on the highest capacity 151 mAh g^{-1}.

Supplementary Table 3. Comparison of state-of-art multivalent metal-ion batteries. (Dual ion and hybrid systems are therefore not included.) Calculation is based on the mass of cathode active materials at 25 °C unless otherwise indicated.

Cathode material	Anode	Current density (mA g^{-1})	Cell voltage (V)	1st discharge capacity (mAh g^{-1})	Capacity retention	Cycling stability (cycle number)	Ref
$MnFe(CN)_6$	Ca-Sn*	10	1.5	85	50%	35	29
Ca_xMoO_3	Ca	10	1.0	100	80%	3	23
VS_4	Ca	100	1.8	316	21%	20	25
S_8	Ca	167	2.1	760	16%	15	24
PAQS	Ca	113	2.1	169	66%	6	27
14 PAQ	Ca	260	2.1	253	63%	4	This work
14 PAQ	Ca_xSn	130	1.8	248	61%	1200	
		260	1.8		31%	5000	
14 PAQ	$CaSn_3$	130	1.5	202	29%	1000	
Mo_6S_8	Mg	0.1 mA cm^{-2}	1.1	75	85%	2000	1
Ti_2S_4 (60 °C)	Mg	24	1.2	195	72%	40	2
PTO	Mg	81.6	2.0	315	84%	700	36
14 PAQ	Mg	260	1.3	221	37%	1000	37
S_8	Mg	167	1.2	820	69%	110	3

* The Ca-Sn anode was prepared by electrochemically calciation of a Sn electrode.

Supplementary References:

1. Aurbach, D. *et al.* Prototype systems for rechargeable magnesium batteries. *Nature* **407**, 724–727 (2000).
2. Sun, X. *et al.* A high capacity thiospinel cathode for Mg batteries. *Energy Environ. Sci.* **9**, 2273–2277 (2016).
3. Gao, T. *et al.* Reversible S^0/MgS_x Redox Chemistry in a $MgTFSI_2/MgCl_2$/DME Electrolyte for Rechargeable Mg/S Batteries. *Angew. Chemie* **129**, 13711–13715 (2017).
